# Construction of Silk Fibroin 3D Microfiber Scaffolds and Their Applications in Anti-Osteoporosis Drug Prediction

**DOI:** 10.3390/molecules29235681

**Published:** 2024-11-30

**Authors:** Hua Xu, Mengfan Huang, Mengyuan Zhou, Rong Guo, Kunming Qin, Zibo Dong

**Affiliations:** 1 School of Pharmacy, Jiangsu Ocean University, Lianyungang 222005, China; 2020220656@jou.edu.cn (H.X.); 2022221109@jou.edu.cn (M.Z.); 2022221022@jou.edu.cn (R.G.); 2 Southern Medical University, Guangzhou 511453, China; hmfan1@126.com

**Keywords:** NaBH_4_ degumming, 3D scaffold, TFSC, drug loading

## Abstract

Silk microfiber scaffolds have garnered increasing interest due to their outstanding properties, with degumming being the process used to extract the sericin from the cocoon. In the present study, an attempt to tune the biodegradation period of silk through degumming with various sodium borohydride (NaBH_4_) concentrations and degumming times was studied. We considered the process, the number of baths used, and the salt concentration. Herein, we report a novel method of expanding microfibers from two-dimensional (2D) to three-dimensional (3D) using a modified gas-foaming technique. Porous three-dimensional (3D) silk fibroin (SF) scaffolds were fabricated by the SF fibers, which were extracted by the NaBH_4_ degumming method and NaBH_4_ gas-foaming approach. This study showed that higher salt concentrations, reaching 1.5% in a double bath, effectively removed sericin from silk fibroin, resulting in clean, smooth 3D scaffolds. These scaffolds were then fabricated using a freeze-drying method. The scaffolds were then submerged in solutions containing semen cuscutae (SC) and their surfaces were coated with various percentages of total flavonoids. The scaffolds had no toxicity to the cells in vitro. This work provides a new route for achieving a TFSC-loaded scaffold; it is proved that the coated silk fibroin fiber scaffold has excellent compatibility. Compared with non-drug-loaded silk scaffolds, drug-loaded silk scaffolds promote cell growth.

## 1. Introduction

Osteoporosis is a prevalent bone disease that significantly increases the risk of fractures, along with delayed bone healing and impaired bone regeneration. Globally, over 200 million individuals are affected by osteoporosis [1,2]. In osteoporotic conditions, excessive osteoclast activity leads to decreased bone density and quality, which compromises the supporting capacity of orthopedic implants and hinders implant fixation [3]. Consequently, the development of scaffolds that exhibit strong osteogenic activity and high-quality osteogenesis is crucial for enhancing the repair of osteoporotic bone, thereby reducing complications and associated healthcare costs [4]. Associating inductive factors with scaffolds represents a promising strategy for reconstructing lost bone. However, the high cost and limited active duration of growth factors pose significant challenges to their broader clinical application. As a potential alternative, traditional Chinese medicine has garnered increasing interest due to its low production costs and multi-component therapeutic properties [5]. *Cuscuta australis* R.Br is classified as a member of the morning glory family in older references. Total flavonoids from semen cuscutae (TFSC), are the primary estrogenic active component of the plant and are one of the most commonly used drugs for osteoporosis. TFSC are believed to be effective in bone repair in bone defects including osteoporosis in traditional Chinese medicine. Research has shown that total flavonoids derived from semen cuscutae can have beneficial effects on bone formation by promoting the osteogenesis of bone mesenchymal stromal cells (BMSCs) and inhibiting the activity of osteoclasts [6].

**Figure 1:**
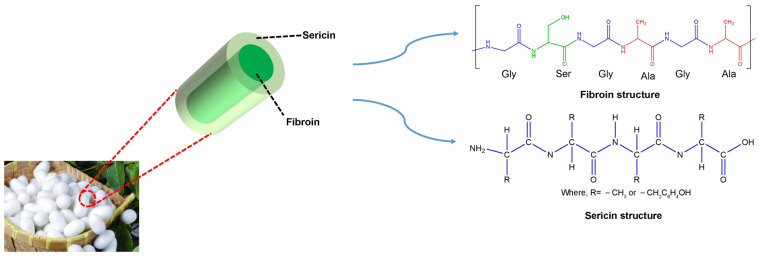
The chemical structure of silk fibroin and silk sericin.

An ideal bone scaffold must be able to accelerate bone healing and impaired bone regeneration. One material that may be used and modified in scaffold manufacturing for tissue engineering is silk due to its molecular composition and physical and chemical structures. Silk is a naturally occurring polymer of protein fibers, referred to as the queen of textiles and the silk textile industry, produced by the silkworm Bombyx mori. The protein fibers are composed of sericin and fibroin; the chemical structure of silk proteins is shown in Figure 1. The biocompatibility and biodegradability of silk have been questioned with the rapidly increased application [7,8]. Many studies reported compatibility issues of SF due to the presence of silk sericin (SS) and further research demonstrated that residual sericin can cause a serious allergic reaction [9]. SS surrounds the exterior of silk fibers; SS is removed through the degumming process. The aim of the degumming process is to improve the mechanical properties and to improve the compatibility. Traditional degumming methods have been adopted, such as alkali, acid, foam, microwave, and enzyme [10]. However, these degumming methods may be shadowed by limitations, including costly equipment, high energy consumption, and high operating costs. As a result, it is highly meaningful to develop a new approach for silk degumming with improved fiber quality and a decrease in the strength of the fibers. In this study, a unique silk degumming approach was adopted by NaBH_4_, which was considered a potential source of hydrogen due to its non-toxic property and high hydrogen content. Previously, the expansion of 2D fibers into 3D structures using gas bubbles generated by chemical reactions in NaBH_4_ aqueous solutions was studied [11,12,13,14]. In this study, a combination of the degumming approach and gas-foaming approach with NaBH_4_ solutions was used to fabricate silk fibroin 3D microfiber scaffolds.

NaBH_4_ is a complex compound with strong reducibility. At the same time, NaBH_4_ has the characteristics of high hydrogen energy storage, convenient hydrogen release, and relatively stable chemical properties, which makes NaBH_4_ aqueous solution slowly react with water to release hydrogen [15,16,17]. A large number of hydrogen bubbles get through the silk fiber. Under the action of inertial force and surface tension, hydrogen bubbles along the silk fiber produce an upward pull on the sericin on its surface. Because sericin contains a high concentration of L-arginine and L-lysine structures, the peptide bond between an L-arginine or L-lysine residue and another amino acid residue is initially broken down by hydrogen bubbles.

An ideal scaffold should consist of hierarchically assembled fibers with controlled alignments. Some research shows that the fibers intentionally oriented in 3D can significantly guide cell migration into the scaffold in vitro and in vivo [12,13].

Some studies that have been conducted clearly demonstrate that electrospun nanofiber mats can be expanded in the third dimension (3D) after treatment with aqueous gas bubble chemical reactions in an aqueous solution [12]. The findings of this study aim to provide insight into the design of 3D biomimetic scaffolds for bone tissue repair and regeneration in situ and engineering 3D bone models in vitro.

TFSC with high bioactivity and fewer side effects are used for bone repair. However, there are drawbacks, such as them being drugs of immediate release. In this study, we combined total flavonoids from semen cuscutae, which can significantly stimulate bone regeneration, with novel microfiber scaffolds. We designed these scaffolds with controllable pore sizes and complex structures to facilitate cell migration and proliferation while also promoting the efficient diffusion of total flavonoids for tissue regeneration.

## 2. Results and Discussion

### 2.1. Degumming Analysis

**Figure 2:**
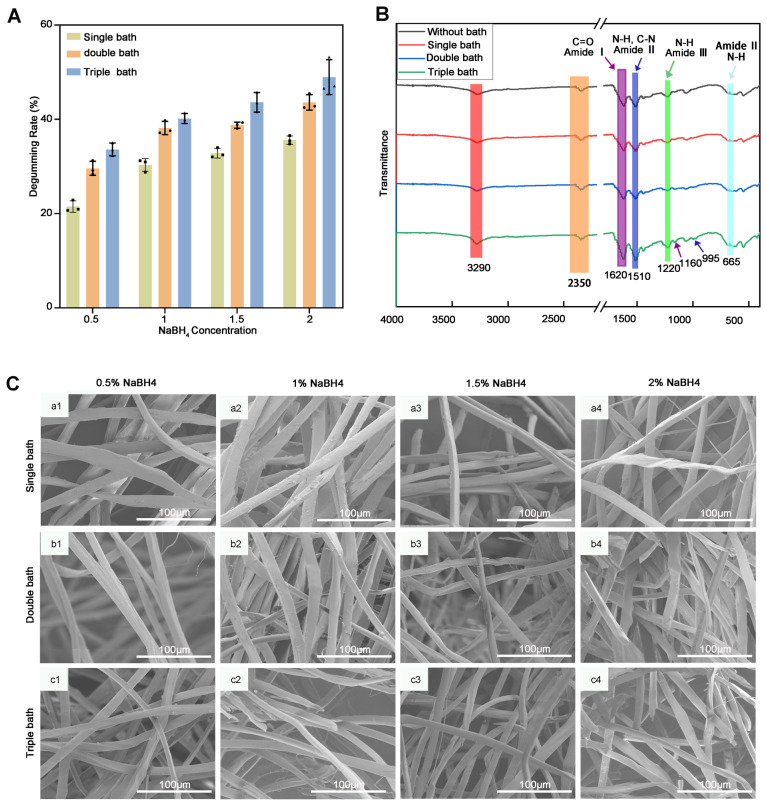
(**A**) The degumming ratio of silkworm cocoons treated with varying concentrations and varying baths; (**B**) FTIR spectra of silkworm cocoons and degummed silk fibers (1% NaBH_4_, 12 h); (**C**) SEM micrographs of the fibers from the 12 degumming treatments, (**a1**–**a4**) single bath, (**b1**–**b4**) double bath and (**c1**–**c4**) triple bath.

Degumming analysis of degummed silk fibers was performed using the degumming rate and Equation (1), mentioned in the previous section. The cocoon degumming rate at room temperature increased from 0.5% to 2%; the degumming rate increased from the first bath to the third bath, as shown in Figure 2A.

Scanning electron microscopy (SEM) was employed to observe the microscopic morphological changes of the samples across different concentrations and baths, allowing for an analysis of the relationship between these morphological changes and the processing methods used. The SEM images of silk fibers presented a clear view of the degumming capability. In general, the residual sericin in silk fibroin was significantly reduced by increasing the NaBH_4_ concentration or going from single bath, to double bath, to triple bath in Figure 2C.

Furthermore, degummed silk was analyzed using FTIR analysis for confirmation of the sericin removal in Figure 2B. FTIR analysis of the silk fibers (without bath, single bath, double bath, and triple bath) showed inherent peaks at 1650–1630 cm^−1^ for amideⅠ(C=O stretching), 1530–1500 cm^−1^ for amide II (secondary N-H bending), 1270–1230 cm^−1^ for amide III (C-N and N-H functionalities), and 750–600 cm^−1^ for amide Ⅱ in the FTIR spectra. FTIR analysis of silk fiber revealed characteristic inherent peaks at 3290 cm⁻^1^ and 1220 cm⁻^1^, which correspond to the Amide-A group, Amide-III group, and in-plane ‘=C-H’ group vibrations, respectively. These peaks are commonly found in silk fibroin [18]. Additionally, strong peaks observed at 1620 cm⁻^1^ and 1510 cm⁻^1^ indicate the presence of the Amide-I group (C=O stretch) and the Amide-III group (C-N stretch, N-H bend), which are also typically associated with silk fibroin. Furthermore, peaks at 1160 cm⁻¹ and 995 cm⁻^1^ in silk fibers subjected to a triple bath confirm the effective removal of sericin from the silk fibers.

### 2.2. Fabrication and Characterization of 3D Silk Microfiber Scaffolds

**Figure 3:**
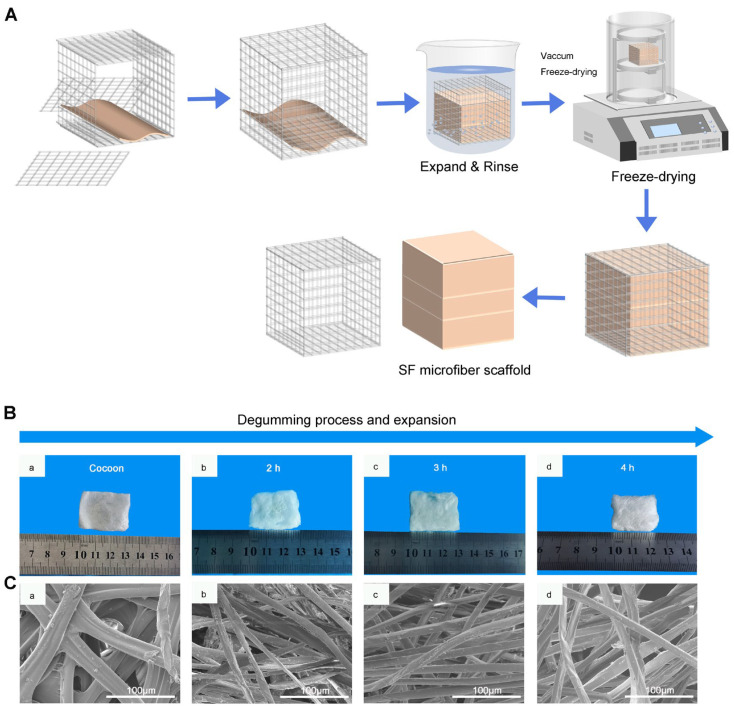
Preparation of SF 3D microfiber scaffolds. (**A**) Schematic illustration of the fabrication of expanded SF microfiber scaffolds. (**B**) Photographs showing the silk scaffolds before and after treatment with 1% NaBH_4_. (**a**) Without Soaking; (**b**) Soaking in NaBH_4_ solutions for 2 h; (**c**) Soaking in NaBH_4_ solutions for 3 h; (**d**) Soaking in NaBH_4_ solutions for 4 h. (**C**) Morphology of silk microfiber scaffold SEM images before and after treatment with 1% NaBH_4_. (**a**) Without Soaking; (**b**) Soaking in NaBH_4_ solutions for 2 h; (**c**) Soaking in NaBH_4_ solutions for 3 h; (**d**) Soaking in NaBH_4_ solutions for 4 h.

For the fabrication methodology, we first cut the silkworm Bombyx mori into desired mats, as described in our previous studies [13]. We have described a robust approach for the fabrication of expanded microfiber scaffolds with a precise control of thickness by using a customized iron wire mold, as shown in Figure 3A.

Figure 3B shows the photographs of expanded microfiber mats and freeze-dried, expanded microfiber scaffolds’ thickness increasing as the time of the microfiber mats immersed in the NaBH_4_ solution was prolonged.

Figure 3C shows a scanning electron microscopy (SEM) image of the microfiber scaffolds. The residual sericin in silk fibroin was significantly reduced by extending the soaking time. Nevertheless, a significant amount of residual sericin was retained on the fiber surface.

### 2.3. In Vitro Degradation Behavior of Degummed Silk

#### 2.3.1. Morphology

**Figure 4:**
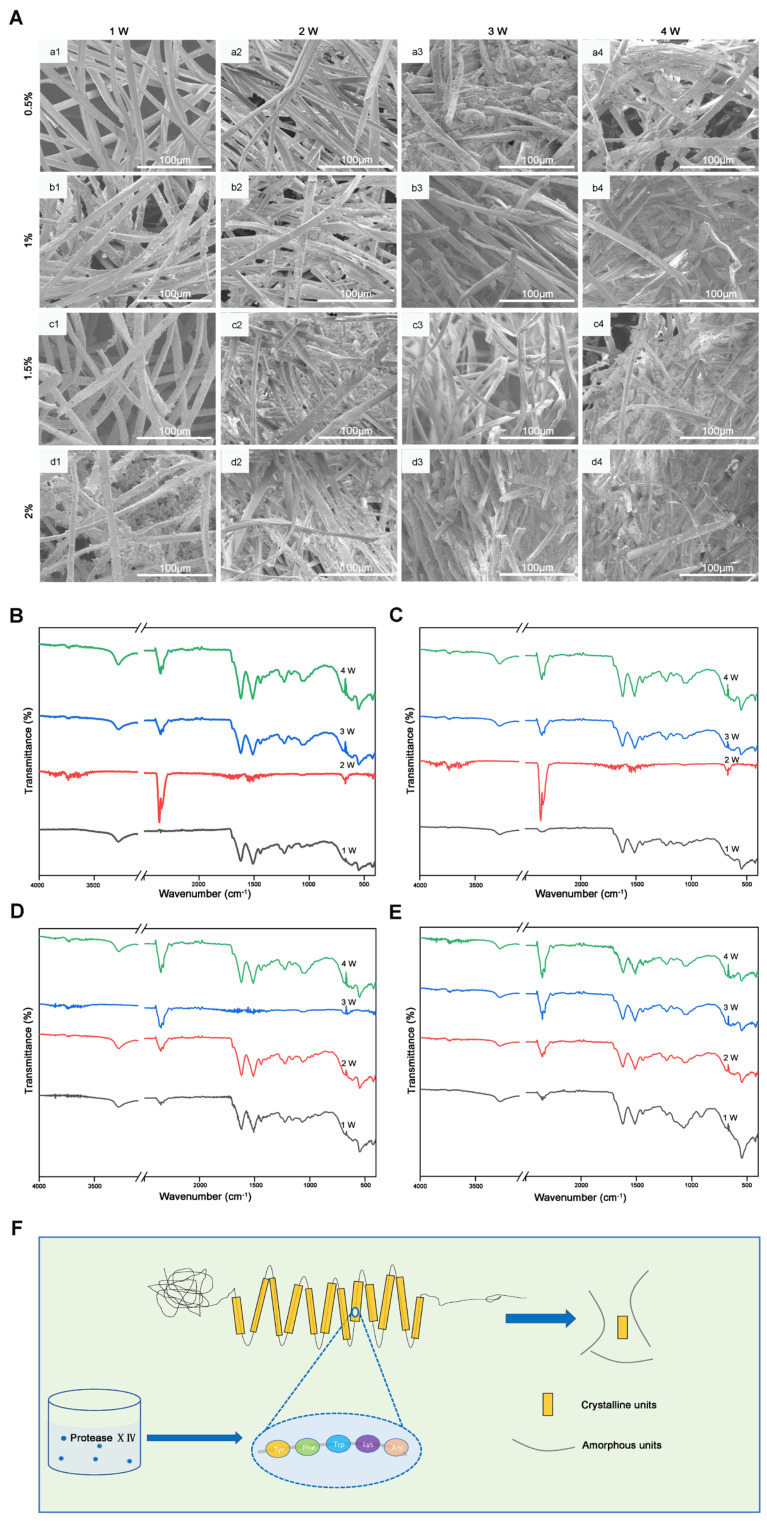
In vitro degradation behavior of degummed silk scaffold. (**A**) SEM images of degummed silk after enzymatic degradation with time; (**a1**–**a4**) 0.5% NaBH_4_, (**b1**–**b4**) 1% NaBH_4_, (**c1**–**c4**) 1.5%NaBH_4_, (**d1**–**d4**) 2% NaBH_4_. (**B**) 0.5% NaBH_4_, 12 h, (**C**) 1% NaBH_4_, 12 h, (**D**) 1.5% NaBH_4_, 12 h, (**E**) 2% NaBH_4_, 12 h, FTIR spectra of degummed silk scaffolds after enzymatic degradation with time. (**F**) Plausible mechanism for the degradation of SF scaffolds.

The integrity of the degummed silk after enzymatic degradation over a 28-day period was studied using scanning electron microscopy (Figure 4A). The electron microscopy results indicated that silk fibers degummed with 0.5% NaBH_4_ displayed a flat surface after 7 days of enzymatic treatment. This was followed by a stepwise increase in fibrillation and the exposure of more filaments as the enzymatic treatment time increased. Observations revealed that the separated filaments retained a smooth surface and intact structure, signifying only slight damage to the fibrillar structure of the native silk. A few filaments were noted in the silk samples as degummed with 0.5%, 1%, 1.5%, and 2% NaBH_4_ following enzymatic degradation.

#### 2.3.2. Structural Changes in Degraded Silk

Fourier Transform Infrared (FTIR) analysis was performed to evaluate the silk fibroin (SF) scaffolds before and after enzymatic degradation, as illustrated in Figure 4B–E. Native silk is characterized by its highly crystalline fibroin structure; however, the degraded silk samples exhibited significant absorption peaks around 1620 and 1515 cm^−1^, along with a shoulder peak at 1260 cm^−1^. These peaks are associated with the β-sheet conformation, indicating that the predominant silk II structure of the fibers remained unchanged. The similarity of the characteristic peaks in the spectra of the degraded silk confirms that the degumming process did not cause any damage or alterations to the silk’s secondary structure.

The degradation mechanism can be attributed to the actions of protease enzymes found in the proteolytic culture and cow dung, which specifically target the amorphous random coil regions of silk fibroin. The proteolytic culture used in the degradation process contains various serine proteases, including Proteinase K and Protease XIV. Protease XIV cleaves peptide bonds near hydrophobic, aromatic, and aliphatic amino acids. Silk fibroin, sourced from the cocoons of Bombyx mori, primarily consists of a light chain, a heavy chain, and a P25 glycoprotein [19,20]. The heavy chain comprises 11 hydrophilic domains and 12 hydrophobic domains [21], with the latter containing β-sheet regions characterized by repetitive amino acid motifs, particularly glycine-X units, where X can be serine, alanine, or tyrosine. Predictive analyses by Brown et al. and Wongpinyochit et al. have identified numerous cleavage sites within the heavy chain of silk fibroin for the enzymes Protease XIV and Proteinase K. Specifically, the predicted cleavage sites were 390 for Protease XIV and 2200 for Proteinase K, irrespective of the accessible surface area [22,23]. The proposed mechanism for silk fibroin degradation using proteolytic culture is depicted in Figure 4F.

### 2.4. The Highlights of the In Vivo Degradation Behavior of Degummed Silk Fibers

**Figure 5:**
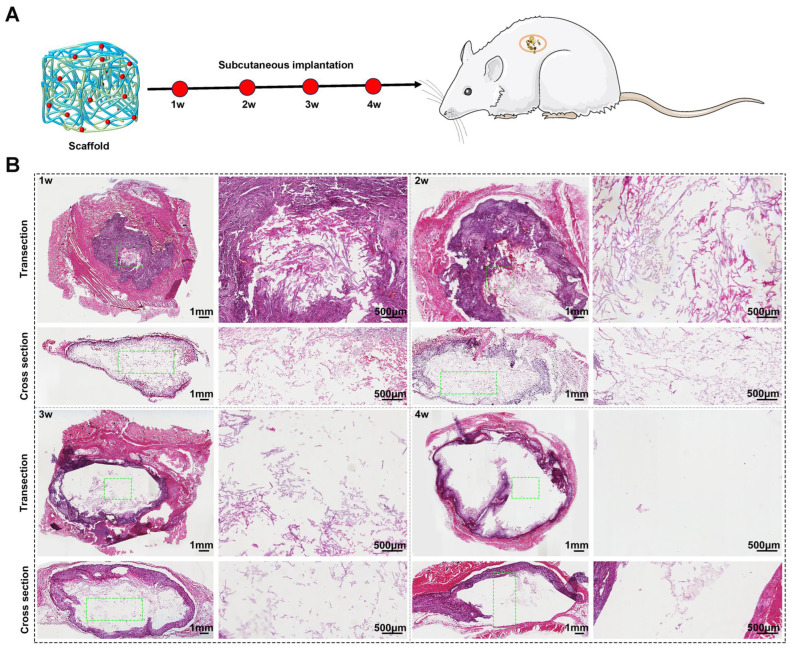
In vivo degradation behavior of the degummed silk scaffold. (**A**) Schematic illustration of subcutaneous implantation of the expanded 3D silk microfiber scaffold; (**B**) Histological image of H&E staining of the subcutaneously implanted silk fiber scaffold at 1 week, 2 weeks, 3 weeks, and 4 weeks. Green square indicate enlarging some areas of the implanted silk scaffold.

Hematoxylin and eosin (H&E) staining was performed to further investigate the degradation behavior and tissue response of degummed silk fibers in vivo. All types of degummed silk fibers exhibited partial degradation, with no significant signs of infection observed. After two weeks, the wounds showed signs of re-epithelialization and granulation tissue formation, indicating a good state of repair. The implanted silk fibers were harvested at Postoperative Weeks 1, 2, 3, and 4 (Figure 5A). Figure 5B presents the histological sections of degummed silk fibers after subcutaneous implantation, stained with H&E. One week post-implantation, the silk fibers were rapidly encapsulated by connective tissue, with degradation occurring from the interface between the tissue and the implant, progressing inward. A substantial number of cells infiltrated and aggregated around the silk fibers (Figure 5B) and, in addition to the cellular and fibrous tissue ingrowth, evident vascularization was observed surrounding the silk fibers.

### 2.5. HPLC-PAD Fingerprints of Decoction

**Figure 6:**
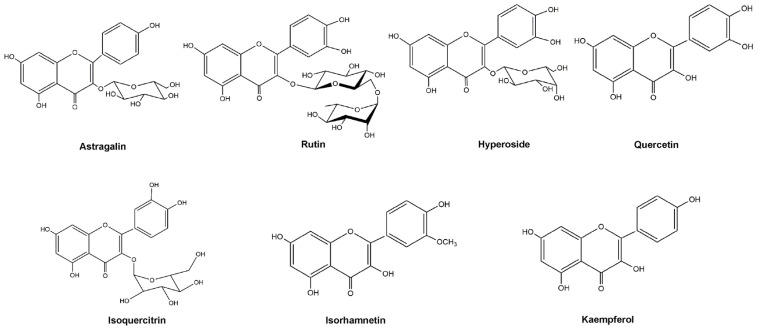
TFSC structure.

**Figure 7:**
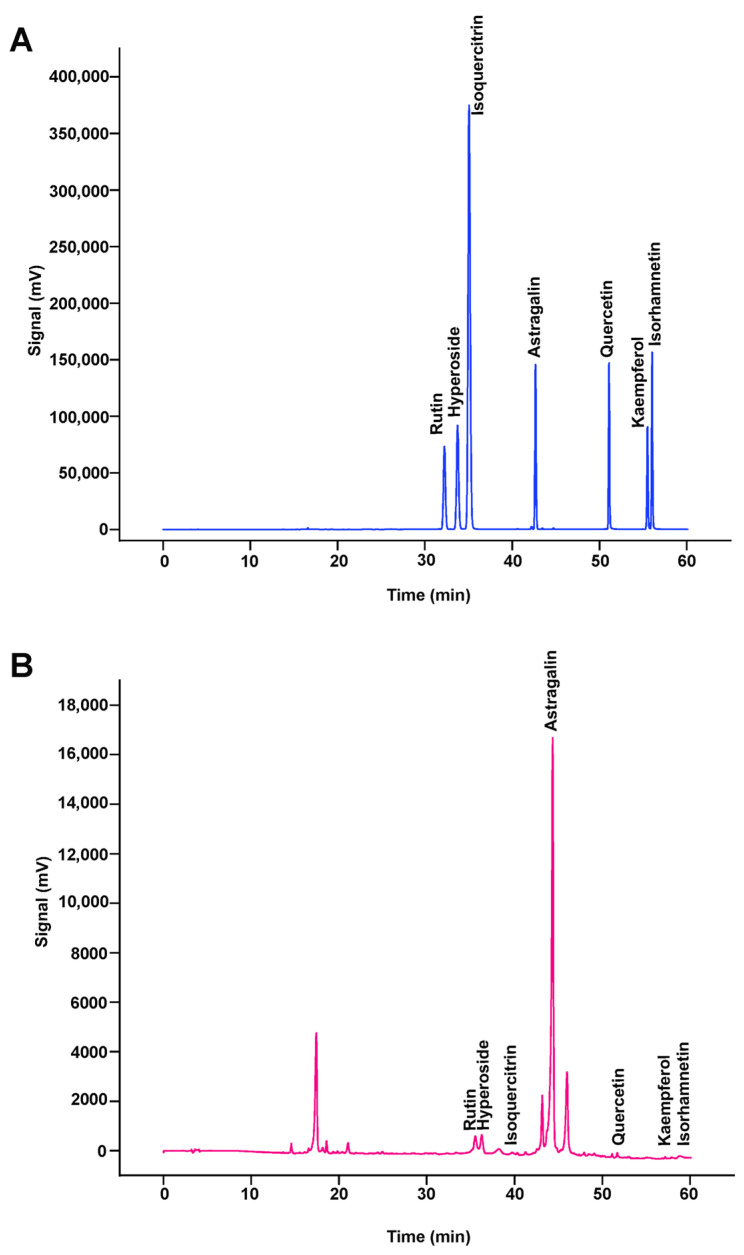
The HPLC fingerprints of the mixed standard substance (**A**) and TFSC samples (**B**).

Samples of total flavonoids from semen cuscutae solutions and standard solutions were injected into high-performance liquid chromatography (HPLC) to obtain chromatograms (Figure 6). The fingerprint profiles of the decoction were generated through analysis and processing, resulting in the calibration of a total of seven common peaks and the identification of eight components, with specific data presented in Figure 7.

### 2.6. Fabrication and Characterization of SF 3D Microfiber-Coated Scaffolds

**Figure 8:**
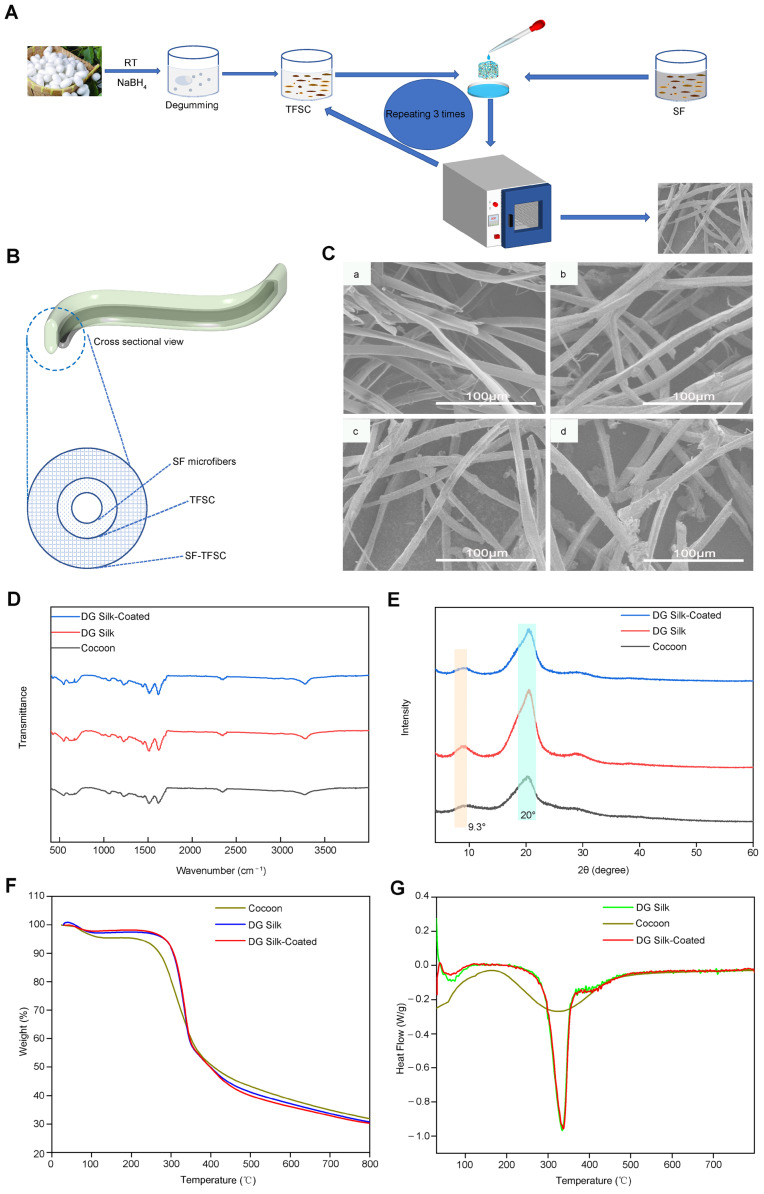
Schematic illustration of the fabrication and characterization of SF 3D microfiber-coated scaffolds. (**A**) Schematic diagram of the fabrication of 3D microfiber-coated scaffolds; (**B**) The cross-sectional view of SF 3D microfiber-coated scaffolds; (**C**) SEM images of 3D microfiber-coated scaffolds: (**a**) NO group: scaffold samples without coating; (**b**) TFSC-SF-1 group: The degummed microfibers scaffolds were soaked in 1 mg/mL TFSC solution and then dipped in a solution containing 4 mg/mL TFSC, 7% SF at a volume ratio of 1:10; (**c**) TFSC-SF-2 group: The degummed microfibers scaffolds were soaked in 4 mg/mL TFSC solution and then dipped in a solution containing 4 mg/mL TFSC, 7% SF at a volume ratio of 1:10; (**d**) TFSC-SF-3 group: The degummed microfibers scaffolds were soaked in 7 mg/mL TFSC solution and then dipped in a solution containing 4 mg/mL TFSC, 7% SF at a volume ratio of 1:10; (**D**) FTIR spectra of DG silk-coated scaffolds, DG silk scaffolds and cocoon; (**E**) XRD spectrum of DG silk-coated scaffolds, DG silk scaffolds and cocoon; (**F**) Thermogravimetric (TG) curves of DG silk-coated scaffolds, DG silk scaffolds and cocoon; (**G**) Derivative thermogravimetric (DTG) curves of DG silk-coated scaffolds, DG silk scaffolds and cocoon.

As shown in Figure 8A,B, coating is usually used for scaffolds. Bioactive substances with certain functions, such as drugs, are applied to the coating of scaffolds to sustain drug release and bone repair [24]. The coating treatment can reduce the friction resistance during scaffold implantation. Hence, the smooth progress of the operation can reduce the pain of the patient.

As shown in Figure 8C, the surface of the uncoated scaffold and coated scaffolds (TFSC-SF-1, TFSC-SF-2) are smooth while the coated scaffolds (TFSC-SF-3) have a rough surface.

ATR-FTIR spectroscopy was performed to analyze the structure of the SF degumming and SF coatings. The FTIR spectra of the silkworm Bombyx mori, the degummed silk microfibers, and the coated silk microfibers (TFSC-SF-2) are shown in Figure 8D [25]. The absorption bands at 1510 cm^−1^ and 1440 cm^−1^ are characteristic of the SF that corresponds to the amide A linkage; C=O stretching by the carboxylic groups, as well as N-H; and bending present in Amide-III, respectively. The peaks present in 1160 cm^−1^, 670 cm^−1^, and 554 cm^−1^ correspond to the C-H stretching of sericin proteins. As shown in Figure 8D, the curves of the degummed silk fibers and the coated silk fibers present an absorbance peak of around 670 cm^−1^ and 554 cm^−1^. It means the coated silk fibers do not impact the secondary structure of SF.

In Figure 8E, the XRD spectrum shows clear diffraction peaks at 9.3° and 20°. XRD analysis was performed for the silkworm Bombyx mori, the degummed silk microfibers, and the coated silk microfibers to validate the conformation of SF microfibers. A diffraction peak pattern observed at 20° and 9.3° was attributed to the β-sheet crystalline structure of silk. It was examined that all silk microfibers exhibited a broad peak at 2θ = 9.3° or 20°, indicating the main silk II crystal structure, which comprises all the natural patterns of amorphous silk materials. The observation arising from the XRD spectrum is in accordance. The SF structure is mainly composed of the β-sheet structure and its peptide chains are arranged neatly. Intermolecular attraction and hydrogen bond tightly.

Figure 8F,G illustrate the thermal stability of various silk fibers, characterized by thermogravimetric analysis (TGA) and derivative thermogravimetric analysis (DTG), respectively. Figure 8F shows that as the temperature increased, the weight of all silk fibers gradually decreased, with the onset of decomposition beginning at 250 °C and the rate of weight loss rapidly increasing. This weight loss is likely due to the elimination of absorbed water. Figure 8G indicates that the endothermic transition for all silk fibers occurred around 330 °C, attributed to the thermal decomposition of the silk fibers. Although the trends for all silk microfibers were similar, it was observed that the rate of decrease for silkworm (Bombyx mori) silk was lower than that of the other silk fibers, potentially due to the initial decomposition temperature of silk sericin. Regarding SF, from room temperature to approximately 65 °C, water evaporation accounts for the initial weight reduction in SF fibers. The second stage of weight loss, occurring between 250 °C and 335 °C, may be due to the degradation of the silk fiber’s main chain. Figure 8G also illustrates that the residual mass of the cocoon was relatively larger than that of other silk fibers, likely due to its susceptibility to sericin. The thermal stability of the coated silk microfibers was found to be similar to that of degummed silk fibers.

### 2.7. Cell Cytotoxicity

**Figure 9:**
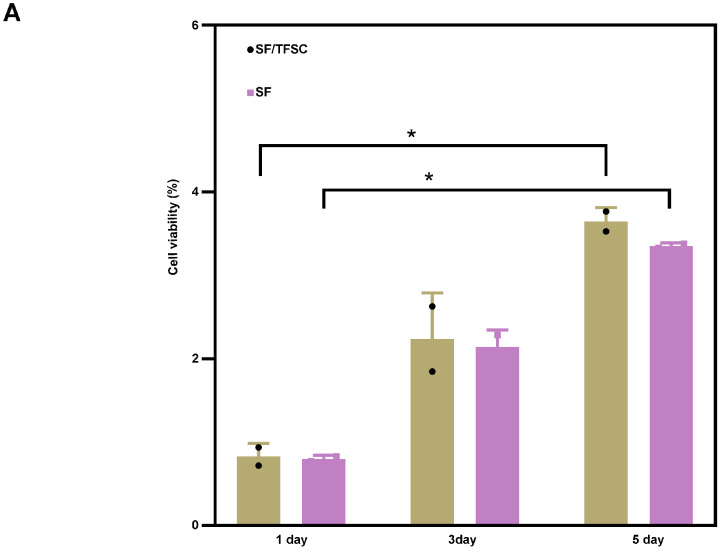

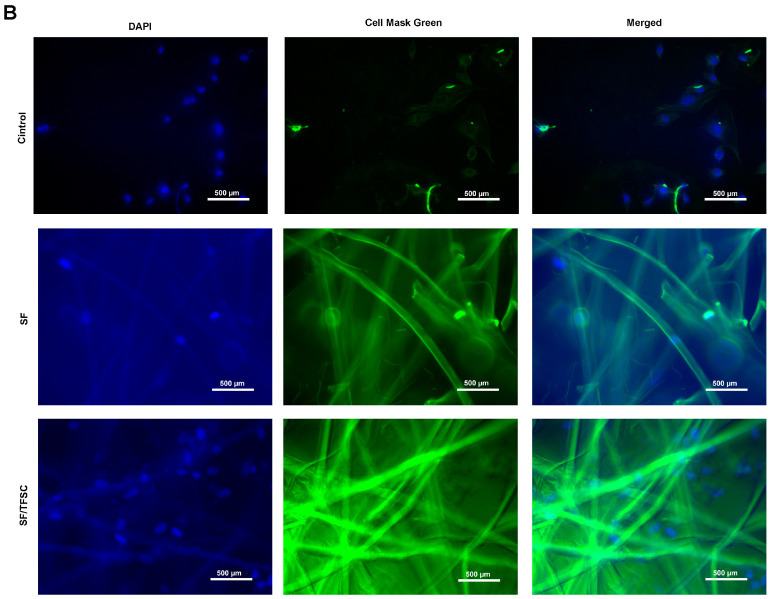
Cell viability investigation. (**A**) CCK-8 assay result of MC 3T3-E1 cell proliferation after 1, 3, and 5 days, * indicate significance; (**B**) Morphology of MC 3T3-E1 cells under the effect of the control, SF, and SF/TFSC.

The silk microfiber scaffolds were evaluated for cell proliferation using CCK-8 staining (Figure 9B). Cell proliferation at 1, 3, and 5 days met the survival requirements according to [26] International Organization for Standardization. ISO 10993, ISO, 2003, where a cell survival rate greater than 75% is considered non-cytotoxic. Thus, all samples were deemed non-cytotoxic (Figure 9A). MC 3T3-E1 cells were cultured in SF and TFSC/SF scaffolds for the indicated days, respectively. With the extension of experimental time, the viability of MC 3T3-E1 cells on scaffolds was enhanced continuously, On the third day, no significant difference in MC 3T3-E1 cell proliferation was found between the scaffolds. On the fifth day, TFSC/SF scaffolds significantly increased the proliferation of the MC 3T3-E1 cultured on it. As shown in Figure 9B, after removing the culture medium and repeatedly washing cells, we stained the nucleus with blue 4′,6-diamidino-2-phenylindole (DAPI) and the cytomembrane with Cell Mask Green, respectively. According to the results (Figure 9B), the MC 3T3-E1 cell number of each sample increased gradually at each time point. However, TFSC/SF scaffolds had the largest number of live cells. These results were in agreement with the CCK-8 staining for cell proliferation.

## 3. Materials and Methods

### 3.1. Materials

Partially degummed natural silk hanks from the Chinese silkworm Bombyx mori were purchased from the Jiangnan Sericulture Base, Jinhua, China. Distilled water (DI) was prepared in the laboratory. NaBH_4_ was purchased from Shanghai Lingfeng Chemical Reagent Co., Ltd., Shanghai, China. Lithium bromide (LiBr, 99%) was obtained from Shanghai Aladdin Biochemical Technology Co., Ltd., Shanghai, China. Total flavonoids from semen cuscutae were provided by Chengdu DeSiTe Biological Technology Co., Ltd., Chengdu, China.

**Figure 10:**
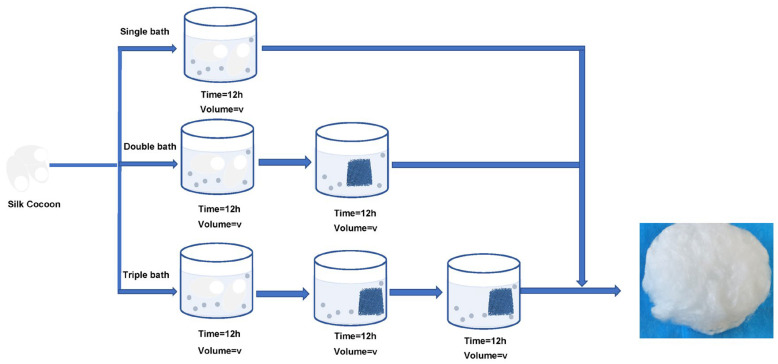
The procedure followed for silk fibroin degumming is outlined in the accompanying scheme.

In their study, Fan et al. [11] utilized NaBH_4_ at varying concentrations (ranging from 0.5% to 2%) for the degumming of silk fibers. It is hypothesized that the generation of H_2_ bubbles occurred gradually over time, leading to a considerable amount of residual sericin remaining on the fiber surface. In the current research, the cocoon underwent degumming at room temperature for 12 h using NaBH_4_ with different concentrations and various bath conditions; a third bath was performed, as graphically described in Figure 10. The best combination of conditions was chosen and compared to varying concentrations and varying baths. The NaBH_4_ concentration variation and the number of degumming baths were studied. After processing, the silk sample was cleaned frequently under pure water and then dried out to measure the weight. The degumming rate was measured. The degumming rate was calculated by the following equation:

Degumming Rate (%) = (W_2_ − W_1_)/W_1_ (1)

where ‘W_2_’ and ‘W_1_’ are the weights of silk before and after degumming, respectively.

### 3.2. Preparation of SF Microfiber Scaffolds

We have described an approach for the fabrication of an expanded microfiber scaffold with a great mat size by using a customized iron wire mold [13,14,27]. We were able to expand microfiber scaffolds with a starting mat size of 30 × 20 mm. Degummed microfiber scaffolds were made after freezing and freeze-drying via direct use of the negative pressure of a freeze-drying machine to make silk fiber fluffy.

### 3.3. In Vitro and In Vivo Degradation of Degummed SF Scaffolds

#### 3.3.1. In Vitro Degradation of Degummed SF Scaffolds

The triple degummed SF scaffolds (0.1 g, *n* = 3 per group and time points) were placed in 5 mL of 1.0 mg/mL Protease XIV in pH 7.4 phosphate-buffered saline (PBS) at 37 °C and replaced the enzyme solutions every day. The degraded SF scaffolds were taken out and washed with PBS water at 7, 14, 21, and 28 days. The degraded microfiber SF scaffolds were collected for the following characterizations.

#### 3.3.2. In Vivo Degradation of Degummed SF Scaffolds

In this study, animal experiments were performed by following the guidelines and procedures approved by the Animal Care and Use Committee of Jiangsu Ocean University. Adult male Sprague-Dawley rats (300–330 g) were randomly divided into 4 groups (*n* = 3 for each group). The rats were anesthetized by injecting 4 wt% chloral hydrate (0.1 mL/kg) and were later fixed on an operating table. The skin was shaved and sterilized for surgery. A 5 mm incision was made on their central backs; the subcutaneous tissue was divided for SF scaffold implantation. Then, the wounds were closed. All rats were housed and fed routinely and the implanted SF scaffolds were harvested at designated time points and fixed with 4% paraformaldehyde overnight at 4 °C, embedded in paraffin, and sectioned at a thickness of 10 μm. The explants were 10 μm thick sections that were deparaffinized, dehydrated, rehydrated, and then underwent hematoxylin and eosin (H&E), followed by observation under light microscopy. The positive area was evaluated according to our previous report [28].

#### 3.3.3. Characterization

The morphology of cocoon, degummed SF scaffolds and degraded silk was observed by scanning electron microscopy (SEM) (Hitachi SU8010, Hitachi Ltd., Beijing, China) after being sputter-coated with gold.

The Fourier Transform Infrared (FTIR) spectra of degummed SF scaffolds and degraded SF scaffolds were obtained using a ATR-IR (Tensor II, Ettlingen, Germany) in the spectral region of 400–4000 cm^−1^.

### 3.4. HPLC Fingerprints of TFSC

#### 3.4.1. Sample Preparation and Standard Solutions

A 0.5 g sample of pulverized powder was accurately weighed into a 20 mL flask, 10 mL 80% methanol was added, and the mixture was sonicated at room temperature for 60 min. The solution was transferred to 25 mL volumetric flasks and then 80% methanol was added to the scale and shaken. The solutions were filtered with a 0.45 μm membrane filter for qualitative analysis. Each standard was accurately weighed and dissolved in methanol to produce a standard stock solution. A mixed standard stock solution was then prepared in methanol from individual standard stock solutions. And various standard solutions were obtained by diluting the stock solutions with methanol to a final concentration of 1.006 mg/mL astragalin, 1.014 mg/mL hyperoside, 1.018 mg/mL quercetin, 1.006 mg/mL rutin, 1.064 mg/mL kaempferol, 1.016 mg/mL isoquercetin, and 1.01 mg/mL isorhamnetin. All the solutions used methanol to obtain a series of working solutions of appropriate concentrations.

#### 3.4.2. Chromatographic Fingerprinting Conditions and Qualitative Analysis

HPLC analysis was carried out on a Shimadzu HPLC system (Shimadzu, Kyoto, Japan) with a PDA detector. The chromatographic separation was performed using a YMC-Pack ODS-C18 column (250 × 4.6 mm, 5 μm) maintained at 30 °C. The detection wavelength was 328 nm with a sample injection volume of 10 μL; the flow rate was 1.0 mL/min. The mobile phase was solvent A (0.1% formic acid in water, *v*/*v*) and solvent B (acetonitrile). The gradient programmer was used according to the following profile: 0–2 min, 5% B; 2–10 min, 5–35% B; 10–30 min, 15–18% B; 30–32 min, 18% B; 32–52 min, 18–45% B; 52–60 min, 45–5% B.

### 3.5. Preparation of SF/TFSC Scaffolds

Hybrid silk-fibroin–gelatin nanofibrous sheets were prepared for drug delivery and regenerative medicine, as previously reported [15]. A L-b-L coating method was used for coating. To increase the drug loading dose of the traditional Chinese medicine on the degummed microfiber scaffolds, the silk microfibers were coated by the TFSC. TFSC solutions include 1 mg/mL, 4 mg/mL, and 7 mg/mL dissolved in ethanol. The outer (second) layer was coated by a SF-based drug loading system, where a SF solution (7%) was mixed with TFSC solution (TFSC-SF) as the outer layer coating for drug-loaded microfibers scaffolds. The second layer was utilized to increase the sustained release of the drug and to improve biocompatibility.

The degummed microfiber scaffolds were divided into four groups according to the different coating methods (Table 1): (a) NO group: scaffold samples without coating; (b) TFSC-SF-1 group: the degummed microfibers scaffolds were soaked in 1mg/mL TFSC solution and then dipped in a solution containing 4 mg/mL TFSC, 7% SF at a volume ratio of 1:10; (c) TFSC-SF-2 group: the degummed microfibers scaffolds were soaked in 4 mg/mL TFSC solution and then dipped in a solution containing 4 mg/mL TFSC, 7% SF at a volume ratio of 1:10; (d) TFSC-SF-3 group: The degummed microfibers scaffolds were soaked in 7 mg/mL TFSC solution and then dipped in a solution containing 4 mg/mL TFSC, 7% SF at a volume ratio of 1:10.

The coating process for the inner layer (without silk fibroin) was conducted for 30 min, followed by rinsing with distilled water. This procedure was repeated twice. The outer layer (with silk fibroin) was applied for 60 min at 25 °C, after which it was rinsed with deionized water and vacuum-dried for 4 h. The morphology and drug release performance of the coated scaffold were evaluated while cytocompatibility was assessed by observing the growth of fibroblasts on the scaffolds (Figure S1).

### 3.6. Characterization and Methods

The SU8010 Field Emission Electron Microscope was used to study the surface. The microfiber scaffolds were characterized using a FTIR spectrophotometer (Tensor II, Bruker, Nehren, Germany). ATR-FTIR spectra were taken in a range of 400–4000 cm^−1^ wavenumbers. The XRD analysis of scaffolds was determined by XRD (D8, Bruker, Nehren, Germany) at 40 kV and 40 mA in a 2θ range of 4–60° with a step of 0.02°. Material thermogravimetric analysis (TGA) was recorded using an analyzer (STA449F3, Netzsch, Selb, Germany) with a nitrogen flow of 30 mL min^−1^ within a temperature range of 25–600 °C and at a heating rate of 10 °C min^−1^.

### 3.7. Cell Cytotoxicity

The cytotoxicity was carried out using the MC 3T3-E1(National Center for Cell Science) that was co-cultured with the scaffold samples in a cell culture medium composed of 10% fetal bovine serum (FBS, Invitrogen Gibco, Waltham, MA, USA), 1% streptomycin-penicillin (Invitrogen Gibco), and α-MEM (Invitrogen Gibco). MC3T3-E1 cells were cultured at a temperature of 37 °C and CO_2_ volume fraction of 5%. Before cell seeding, scaffolds were cut into 10 mg sections, which were placed in 96-well culture plates and then sterilized by γ radiation. The cells were passaged two times, then the cell suspension was cultured to 2 × 10^3^/well and inoculated into 96-well plates containing the different scaffold samples, and the number of cells was then counted. Cell Counting Kit-8 (CCK-8) (Beyotime Biotechnology, Haimen, China) was used to verify the biocompatibility of the scaffolds at 1, 3, and 5 days post-seeding. The inoculated samples were incubated with 0.5 mL of calcein for 20 min at 37 °C and observed under an inverted fluorescent microscope. Live cells emitted green fluorescence. The optical density (OD) of samples was measured using a microplate reader (BioTek, Epoch, Winooski, VT, USA) at 450 nm. And the number of cells on the scaffold was assessed by observing the stable cell proliferation. After the cells were grown on the scaffolds for 24 h, they were washed with PBS, fixed in 4% paraformaldehyde for 15 min, washed three times with PBS, and permeabilized with 0.2% Trition-X-100 for 5 min at room temperature. Subsequently, the cells were blocked with 3% BSA for 10 min at room temperature. After incubation, the treated cells were incubated with a primary antibody that had been diluted with PBS for 1 h at room temperature. After having been washed with PBS containing 0.1% BSA, the cells were incubated with a secondary antibody for 30 min at room temperature. The nuclei and cell membranes of the treated cells were further stained with Cell Mask Green live cell membrane stain solution for 30 min. After three rinses with PBS, nuclei were counterstained with DAPI (4′,6-diamidino-2-fenilindol) solution (1:1000 dilution). Samples were observed and images were captured on a Zeiss laser scanning microscope (Zeiss Group, Oberkochen, Germany).

## 4. Conclusions

The field of scaffold porosity, drug release from scaffolds, and scaffold degradation plays a crucial role in tissue engineering and regenerative medicine. These three factors are closely interconnected and have a significant impact on the success of tissue regeneration and repair. In this discussion, we will provide an overview of the current research in these areas and discuss their importance in the development of effective tissue engineering strategies. Scaffold porosity, TFSC release, and scaffold degradation are key research areas in tissue engineering that are essential for the development of effective regenerative medicine strategies. By understanding and optimizing these factors, researchers can design scaffolds that promote cell infiltration, deliver therapeutic factors, and degrade in a controlled manner to support tissue regeneration and repair. Further research in these areas will continue to advance the field of tissue engineering and bring us closer to realizing the potential of regenerative medicine in clinical applications.

Excessive osteoclast formation and increased bone loss are primary contributors to the incidence of osteoporosis [29]. Many researchers have identified TFSC to prevent bone resorption [30] and we found that TFSC inhibits osteoclastogenesis by reducing ROS production [31,32], suppressing multiple signaling pathways induced by TRAF6 and NOX1. Intervention with TFSC resulted in a decrease in the expression of NFATc1 and C-Fos in bone tissue [33,34,35,36]. TFSC could be easily integrated into silk materials, which makes it appropriate to be used as a drug vehicle.

**Figure 11:**
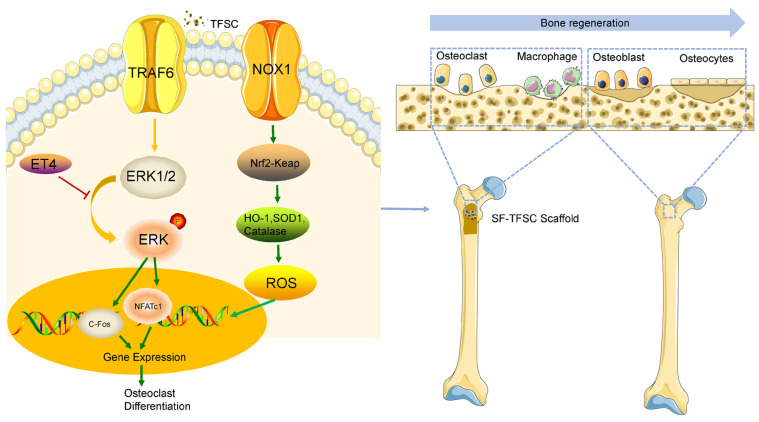
The schematic representation illustrates the mechanism by which TFSC inhibits TRAF6 and NOX1-induced osteoclastogenesis and a schematic illustration of SF 3D microfiber-coated scaffolds for bone regeneration.

Considering the current state of silk and its product fabrication industry, utilizing silk waste to extract SF presents a beneficial approach for resource regeneration and application [37]. We fabricated a novel expanded microfiber scaffold with controlled sizes using a customized mold during a modified gas-foaming process. Additionally, we designed and demonstrated drug-delivery microcarriers based on the expanded microfiber scaffold for anti-osteoporosis activity (Figure 11). The uniform porous microstructures enhance drug loading capacity. It was confirmed that the excellent biocompatibility and rough surface of the SF scaffolds supported normal cell proliferation, which is advantageous for bone repair. These drug-delivery microcarriers based on the expanded microfiber scaffold hold potential for use in bone repairs and regeneration.

## Figures and Tables

**Table 1:** Different study groups of silk microfiber scaffolds with coating methods (shown as contractions and volume ratios) ^a^.

Items	Inner Layer (TFSC)	Outer Layer (TFSC/SF)
NO		
TFSC-SF-1	1 mg/mL	1:10
TFSC-SF-2	4 mg/mL	1:10
TFSC-SF-3	7 mg/mL	1:10

^a^ Note: For this preparation, the SF was prepared at 7 mg/mL.

## Data Availability

Data will be made available on request.

## Supplementary Materials

The following supporting information can be downloaded at: https://www.mdpi.com/article/10.3390/molecules29235681/s1, Figure S1: Drug-release curves of sutures with different concentrations of drug coating methods.
