# Supplementary material for: Construction of Silk Fibroin 3D Microfiber Scaffolds and Their Applications in Anti-Osteoporosis Drug Prediction
Source: Molecules. 2024 Nov 30;29(23):5681. doi: 10.3390/molecules29235681 (PMC11643069; doi:10.3390/molecules29235681)
Supplement: Supplementary file 1 [file molecules-29-05681-s001.zip › molecules-3218729-supplementary.pdf]

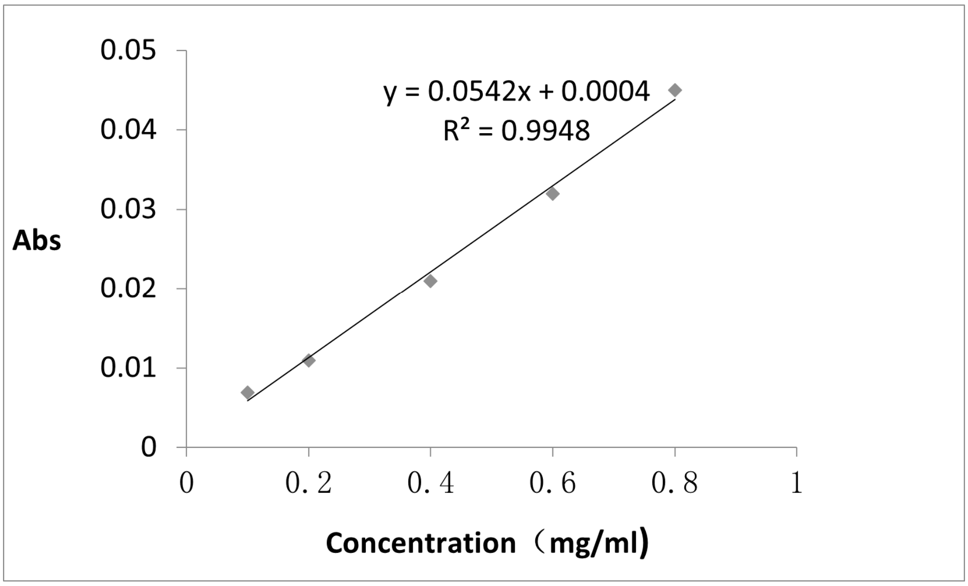

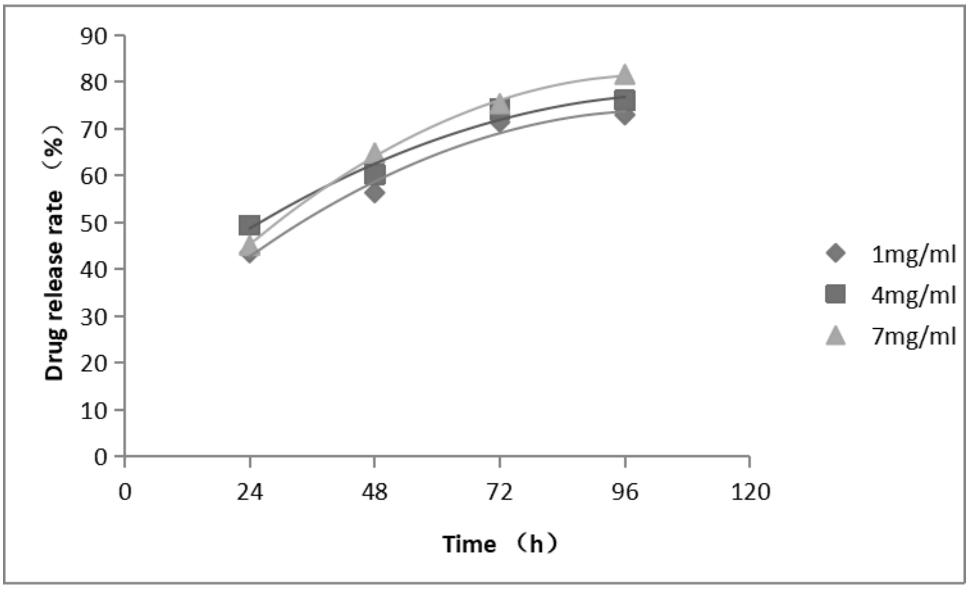

Figure S1. Drug-release curves of sutures with different concentrations of drug coating methods.
